# Analysis of the Stochastic Population Model with Random Parameters

**DOI:** 10.3390/e22050562

**Published:** 2020-05-18

**Authors:** Adeeb Noor, Ahmed Barnawi, Redhwan Nour, Abdullah Assiri, Mohamed El-Beltagy

**Affiliations:** 1 Department of Information Technology, Faculty of Computing and Information Technology, King Abdulaziz University, Jeddah 21589, Saudi Arabia; arnoor@kau.edu.sa (A.N.); ambarnawi@kau.edu.sa (A.B.); 2 Department of Computer Science, Taibah University, Medina 42353, Saudi Arabia; rnour@taibahu.edu.sa; 3 Department of Clinical Pharmacy, College of Pharmacy, King Khalid University, Abha 62529, Saudi Arabia; aalabdullah@kku.edu.sa; 4 Department of Engineering Mathematics and Physics, Engineering Faculty, Cairo University, Giza 12613, Egypt

**Keywords:** population models, stochastic processes, sensitivity analysis, variance decomposition, random parameters

## Abstract

The population models allow for a better understanding of the dynamical interactions with the environment and hence can provide a way for understanding the population changes. They are helpful in studying the biological invasions, environmental conservation and many other applications. These models become more complicated when accounting for the stochastic and/or random variations due to different sources. In the current work, a spectral technique is suggested to analyze the stochastic population model with random parameters. The model contains mixed sources of uncertainties, noise and uncertain parameters. The suggested algorithm uses the spectral decompositions for both types of randomness. The spectral techniques have the advantages of high rates of convergence. A deterministic system is derived using the statistical properties of the random bases. The classical analytical and/or numerical techniques can be used to analyze the deterministic system and obtain the solution statistics. The technique presented in the current work is applicable to many complex systems with both stochastic and random parameters. It has the advantage of separating the contributions due to different sources of uncertainty. Hence, the sensitivity index of any uncertain parameter can be evaluated. This is a clear advantage compared with other techniques used in the literature.

## 1. Introduction

The Verhulst logistic or population equation is a model for many practical applications. It has many applications that imply nonlinear dynamics such as sociology, biology and economics. For example, in epidemiology where the nonlinear term is scaled by a small parameter to denote the contagious rate and in physics to model diffusion with nonlinear perturbations due to unexpected material changes caused by the environment [1].

Deterministic population models have been extensively studied, including models with time variation of the carrying capacity [2,3]. The deterministic models are practical only for sufficiently large populations. Additionally, they neglect many factors that can be significant to the model such as the stochastic and random variations due to different sources.

Population modeling with stochastic variations accounts for the environmental and/or external perturbations in many ecosystems. The environmental fluctuations in the population model can be modeled by adding a noise term [4] or by assuming random variations for one or more parameters e.g., stochastic or random carrying capacity [5].

There are some differences between dynamics and properties of the deterministic and stochastic models. The most important one is the long-time asymptotic dynamics. Other differences include the outbreak probability and the distribution of the final population size [6]. Additionally, the noise term in the population model plays a feedback rule and forces to the system dynamics to stabilize. In the stochastic model, the noise prevents the explosion of the population and is responsible for extinction for one of the species.

The population stochastic differential equation (SDE) is used as a model for the birth–death and population growth processes. As an example, the size of tumor submitted to radiotherapy or phototherapy treatment can be modeled and interpreted mathematically with the population SDE. There are three basic factors affecting the tumor growth. The first is the natural duplication growth of the cells, the second is the self-limitation (may be due to limited available space) of the tumor and the third is due to the treatment. The noise term accounts for the uncertainty due to external influences of environmental factors and/or interaction with other species or due to internal tumor growth [7]. New interesting effects appear due to the presence of the stochastic variations. Randomness introduces more flexibility than the deterministic setting.

Although there is an analytical solution for the 1D population model, the statistical properties are not easily obtained. The problem becomes more complicated for higher dimensions and hence the numerical and spectral techniques should be used. Many numerical techniques based on realizations (sampling) are used to analyze the stochastic population model in 1D and higher dimensions. The numerical techniques are simple to implement but have a slow convergence rate. The Euler–Maruyama (EM) technique has only 0.5 convergence order, while the Milstein (linearized to Runge–Kutta) schemes has convergence of order 1. Some variance-reduction enhancements are done to increase the convergence rate of the numerical techniques. The quasi-Monte Carlo (QMC), as an example, is combined with the tau-leaping technique to enhance the convergence of the stochastic biological models [8], but still the rate is not that efficient. This causes the numerical techniques to be not practical for real applications.

On the other hand, the decomposition Fourier-like techniques such as the Wiener–Hermite expansion (WHE) [9] and the Wiener chaos expansion (WCE) [10,11] have higher orders of convergence. The convergence order of the spectral techniques can be exponential for linear and near-Gaussian processes [12].

For models with random, the polynomial chaos expansion (PCE) motivated by the work of Ghanem and Spanos in 1991 and its generalization (gPC) technique [13] are used efficiently to analyze models with random parameters. They are also efficient techniques in estimating the system sensitivity indices [14,15].

Practically, models that account for both random parameters and stochastic variations are needed for accurate analysis. In the current work, population models with both random parameters and noise variations are considered. The population SDE with random parameters is analyzed with a combination of WHE and gPC decompositions. An equivalent deterministic system is derived and analyzed numerically. The solution statistics, such as mean and variance, are obtained. The effects of the different parameters are quantified, and hence the sensitivity indices are estimated. The results obtained help to understand and control the growth of the population size.

This paper is organized as follows: Section 2 introduces the spectral/decomposition techniques commonly used in the case of noise and random model parameters. Both deterministic and stochastic population models are introduced in Section 3 and Section 4. In Section 5, the stochastic population model with noise imposed is analyzed using WHE. Section 6 extends the stochastic model to have random parameters. A numerical example is analyzed in Section 7 with computations of the sensitivity indices.

## 2. Stochastic Spectral Techniques

Assume that the model contains a set of random parameters U(ω) and depend on a set of n real-valued random variables $\zeta =\left\{\zeta_{1},\cdots ,\zeta_{n}\right\}$ with predefined probability distributions i.e., U(ω)=U(ζ(ω)). Consider the orthonormal basis functionals $\left\{\psi_{k}\left(\zeta \right);\;k\in \mathbb{N}\right\}$ for the space *L^2^* of second-order functionals in the random variables ζ(ω). Many choices for the basis functionals $\psi_{k}$ including multiwavelets or the multivariate polynomials [12]. Any second-order random process X(t, ω) that depends on U(ω) can then be decomposed as follows [16]:

(1) $$X\left(t,\;\omega \right)=\sum_{k\in \mathbb{N}}x_{k}\left(t\right)\;\;\psi_{k}\left(\zeta (\omega )\right).$$

For practical computations, the Expansion (1) is truncated to its first P+1 terms. The gPC expansion converges in the mean square sense and [17], i.e.,

$$\underset{P\to \infty }{\lim}\;{\left(X-\sum_{k=0}^{P}x_{k}\;\psi_{k}\right)}^{2}=0.$$

In the case of the model with Brownian motion randomness, the stochastic process X can be decomposed using (M+1) truncated WHE as follows [9]:

$$X=\overset{M}{\underset{j=0}{\sum }}\int_{-\infty }^{\infty }\int_{-\infty }^{t_{j}}\int_{-\infty }^{t_{j-1}}\cdots \int_{-\infty }^{t_{2}}x_{}^{\left(j\right)}(t_{1},t_{2},\cdots ,t_{j})\;\;dW(t_{1})dW(t_{2})\cdots dW(t_{j})\;.$$

The functionals $x_{}^{\left(j\right)}(t_{1},t_{2},\cdots ,t_{j})$ are usually assumed to be symmetric in the variables $t_{1},\;t_{2},\;\ldots ,\;t_{j}$. But $dW(t_{1})dW(t_{2})\cdots dW(t_{j})=H^{\left(j\right)}(t_{1},t_{2},\cdots ,t_{j})\;dt_{1}dt_{2}\ldots \;dt_{j}$, where $H^{\left(j\right)}$ is the Hermite functional of order *j*. For brevity we shall write $x_{}^{\left(j\right)}$ without its independent variables. Now we can write

(2) $$X=\overset{M}{\underset{j=0}{\sum }}\underset{R^{j}}{\int }\;x_{}^{\left(j\right)}\;H^{\left(j\right)}\;d\tau_{j},$$

where $d\tau_{j}=dt_{1}dt_{2}\ldots \;dt_{j}$ and the integral $\int_{R^{j}}$ is a j-dimensional iterated integral over the variables $t_{1},\;t_{2},\;\ldots ,\;t_{j}$.

The WHE details are explained in many references cited in the current work, especially the contribution in [9] and the references therein. Additionally, below a simple linear stochastic model is analyzed in more details. Consider the stochastic linear transport population model:

$$\frac{dX}{dt}=a\;X\;dt+\lambda \;X\frac{dW}{dt}\;\;\;;\;\;x(0)=x_{0}\;,$$

using WHE Expansion (2) to get

$$\frac{d}{dt}\left(\overset{M}{\underset{j=0}{\sum }}\;\underset{R^{j}}{\int }\;x_{}^{\left(j\right)}\;H^{\left(j\right)}\;d\tau_{j}\right)=a\;\overset{M}{\underset{j=0}{\sum }}\;\underset{R^{j}}{\int }\;x_{}^{\left(j\right)}\;H^{\left(j\right)}\;d\tau_{j}\;+\lambda \;H^{\left(1\right)}\overset{M}{\underset{j=0}{\sum }}\;\underset{R^{j}}{\int }\;x_{}^{\left(j\right)}\;H^{\left(j\right)}\;d\tau_{j}$$

multiply by $H^{\left(0\right)}=1$ and $H^{\left(1\right)}$, respectively, and then apply the expectation to get

$$\frac{dx_{}^{\left(0\right)}}{dt}=a\;x_{}^{\left(0\right)},\;\frac{dx_{}^{\left(1\right)}}{dt}=a\;x_{}^{\left(1\right)}\;+\lambda \;x_{}^{\left(0\right)}\;\delta \left(t-t_{1}\right),$$

and so on in cases where higher-order kernels and moments are required. In this linear case, analytical solutions can be obtained, for example,

$$x_{}^{\left(0\right)}(t)=x_{0}\;e^{a\;t}\;\mathrm{and}\;x_{}^{\left(1\right)}(t,t_{1})=\lambda x_{0}\;e^{-a\;t};\;\;t\geq t_{1}$$

and hence

$$E[X]=x_{}^{\left(0\right)}(t)=x_{0}\;e^{a\;t}\;\mathrm{and}\;Var\left[X\right]=\int_{t_{1}=0}^{t_{1}=t}{\left(x_{}^{\left(1\right)}\right)}^{2}dt_{1}=t{\left(\lambda x_{0}\;e^{-a\;t}\right)}^{2}.$$

## 3. The Deterministic Population Model

**Figure 1:**
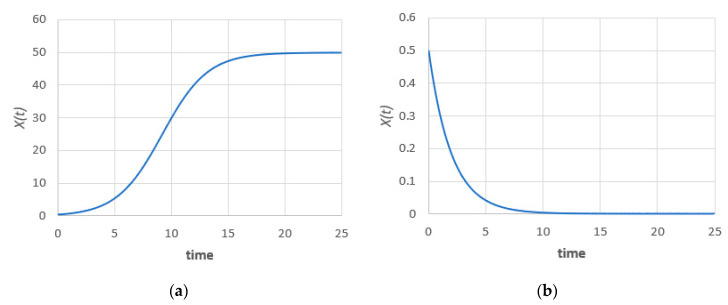
Evolution of the deterministic population for (**a**) case a>0 and (**b**) case a<0.

Consider the transport population model with nonlinear losses:

(3) $$\frac{dX}{dt}=a\;X\;-\;\varepsilon \;X^{2}\;\;\;\;;\;\;\;\;\;x(0)=x_{0}\;\;\;,\;\;\;\;t>0,$$

where the growth rate a=λ−μ, which is the difference between the birth rate λ and death rate μ. The model parameters, a and ε, are assumed deterministic in this case. The model Equation (3) is of Bernoulli type of order 2 and has the exact solution:

$$X(t)=\frac{a\;x_{0}}{\varepsilon x_{0}+\left(a-\varepsilon x_{0}\right)\;e^{-at}}=\frac{L\;}{1+\left(L/x_{0}-1\right)\;e^{-at}},$$

where ε/|a|=1/L is the “crowding term”, the reciprocal of the carrying capacity L (saturation level). For a>0, the system will reach an infinite limit equals the carrying capacity L and the well-known sigmoid/logistic curve is obtained. For a<0, the system decays to zero, i.e., the population will become extinct, as shown in Figure 1. In the current work we shall assume the case of an infinite limit (population persistence) but the techniques presented are also applicable for the population extinction scenario.

A more general form of (3) is called Rechards model [19] and it takes the form

$$\frac{dX}{dt}=a\;X\;-\;\varepsilon \;X^{\beta }\;\;\;\;;\;\;\;\;\;x(0)=x_{0}\;\;\;,\;\;\;\;t>0.$$

Here, the exponent β>0 is called an allometric parameter and it is a measure of curvature. Hence, it can be useful for modeling purposes but will not affect the steady-state behavior. The exact solution is then

$$X(t)=\frac{L\;}{{\left(1+\left(L/x_{0}-1\right)\;e^{-at}\right)}^{\frac{1}{\beta -1}}}.$$

In the current work, we shall consider the original Verhulst model which is Richards model with β=2.

## 4. The Stochastic Model

Consider the stochastic transport population model with nonlinear losses:

(4) $$dX=\left(a\;X\;-\;\varepsilon \;X^{2}\right)dt+\lambda \;dW\;X\;\;;\;\;x(0)=x_{0}\;\;\;,\;\;\;\;t>0,$$

with λ is a diffusion coefficient. In the case of deterministic parameters, a and ε, the noise is the only source of randomness in the model (4). It is more practical to consider the growth rate as a random parameter due to the environmental fluctuations or any other excitations. The nonlinear coefficient ε can be also random for different reasons. In this case, the process X will be under multiple sources of randomness.

In the case of multiple sources of randomness, the process X should locally and globally satisfy the finite variance criteria [18], i.e.,

$$E\left[{\left(X|U\right)}^{2}\right]<\infty ,\;E\left[{\left(X|W\right)}^{2}\right]<\infty \;\mathrm{and}\;E\left[X^{2}\right]<\infty ,$$

where U is the vector of all random parameters in Model (4).

The SDE model (4), with $x_{0}$ > 0, has a unique positive solution X:

(5) $$X(t)=\frac{e^{\left(a\;-\;\frac{1}{2}\lambda^{2}\right)\;t+\lambda \;B(t)}}{\frac{1}{x_{0}}+\varepsilon \int_{0}^{t}e^{\left(a\;-\;\frac{1}{2}\lambda^{2}\right)\;t+\lambda \;B(t)}\;dt},$$

which reduces to the deterministic solution in the case where the diffusion parameter λ=0. The stochastic process X(t) given in (4) and for $a>\lambda^{2}/2$ is recurrent and converges to the stationary probability gamma distribution $\gamma \left(2a/\lambda^{2}-1\;,\;\;\lambda^{2}/2\varepsilon \right)$. For $a<\lambda^{2}/2$, the process decays/converges almost sure to zero [7]. In the case in which $a=\lambda^{2}/2$, a noise-induced transition solution is obtained.

Solution (5) is not helpful in obtaining the exact mean and variance in analytical form. Only approximate analytical formulae can be obtained, see [19] for example. The problem becomes more complicated in the case where one or more of the model parameters is random. In the current work, a numerical technique is introduced that can be used to analyze the stochastic population model even in the case of random variations of the model parameters.

Approximate mean and variance analytical formulae are obtained in [19], but the variance formula is not accurate enough and does not reflect the system dynamics.

## 5. Analysis Using WHE

Use WHE Expansion (2) in SDE (4) to get

$$\begin{array}{ll} \frac{d}{dt}\left(\overset{M}{\underset{j=0}{\sum }}\;\underset{R^{j}}{\int }\;x_{}^{\left(j\right)}\;H^{\left(j\right)}\;d\tau_{j}\right)=a\;\overset{M}{\underset{j=0}{\sum }}\;\underset{R^{j}}{\int }\;x_{}^{\left(j\right)} & H^{\left(j\right)}\;d\tau_{j}\;-\varepsilon \;{\left(\overset{M}{\underset{j=0}{\sum }}\;\underset{R^{j}}{\int }\;x_{}^{\left(j\right)}\;H^{\left(j\right)}\;d\tau_{j}\right)}^{2}\\ & +\lambda \;H^{\left(1\right)}\overset{M}{\underset{j=0}{\sum }}\;\underset{R^{j}}{\int }\;x_{}^{\left(j\right)}\;H^{\left(j\right)}\;d\tau_{j}. \end{array}$$

As it is known in WHE [9], the first (zero-order) term is the mean and the second (first-order) term accounts for the Gaussian part of the stochastic solution. Higher-order terms are the nonGaussian part of the stochastic solution with dominance of the second-order term. So, in most applications, even nonlinear, the second-order WHE is sufficient to capture most of the important system dynamics.

Consider only up to second-order kernel $x_{}^{\left(2\right)}$, apply WHE algorithm to get

(6) $$\frac{dx_{}^{\left(0\right)}}{dt}=a\;x_{}^{\left(0\right)}\;-\;\varepsilon \;\;\left[{\left(x_{}^{\left(0\right)}\right)}^{2}+\int_{R}{\left(x_{}^{\left(1\right)}\right)}^{2}dt_{1}+2\int_{R^{2}}{\left(x_{}^{\left(2\right)}\right)}^{2}dt_{1}dt_{2}\right],$$

(7) $$\frac{dx_{}^{\left(1\right)}}{dt}=a\;x_{}^{\left(1\right)}\;-\;2\varepsilon \;x_{}^{\left(0\right)}x_{}^{\left(1\right)}-\;4\varepsilon \int_{R}x_{}^{\left(1\right)}\left(t_{1}\right)x_{}^{\left(2\right)}\left(t_{1},t_{2}\right)\;dt_{2}+\lambda \;\delta (t-t_{1})\;x_{}^{\left(0\right)},$$

(8) $$\frac{dx_{}^{\left(2\right)}}{dt}=a\;x_{}^{\left(2\right)}\;-\;2\varepsilon \;x_{}^{\left(0\right)}x_{}^{\left(2\right)}-\varepsilon \;x^{\left(1\right)}\left(t_{1}\right)x^{\left(1\right)}\left(t_{2}\right)+\lambda \;\delta (t-t_{2})\;x^{\left(1\right)}\left(t_{1}\right),$$

where δ(.) is the Dirac delta function. The initial conditions, after applying WHE, will be:

$$x_{}^{\left(0\right)}(0)=x_{0},\;x_{}^{\left(1\right)}(0,\;t_{1})=zero\;\mathrm{and}\;x_{}^{\left(2\right)}(0,\;t_{1},\;t_{2})=zero.$$

The system of Equations (6)–(8) can be solved simultaneously using the fixed-point iterative scheme. The linear terms are moved to the left-hand side and are computed at the new time level while the nonlinear terms, and the forcing terms will be in the right-hand side and assumed at the old time level. This requires conditions on the values of ε and λ to obtain a convergent solution.

Explicit numerical solutions can be also obtained. For example, using first-order (in time) finite-difference approximation (FDM) in mean kernel $x_{}^{\left(0\right)}$ Equation (6) to get

(9) $$x_{{}_{i+1}}^{\left(0\right)}=\left(1+a\;\Delta t\right)x_{{}_{i}}^{\left(0\right)}-\varepsilon \;\Delta t\left[{\left(x_{i}^{\left(0\right)}\right)}^{2}+I_{1}+I_{2}\right],$$

where $I_{1}=\int_{R}{\left(x_{}^{\left(1\right)}\right)}^{2}dt_{1}$ and $I_{2}=2\int_{R^{2}}{\left(x_{}^{\left(2\right)}\right)}^{2}dt_{1}dt_{2}$ which are usually small compared with the square of the mean kernel ${\left(x_{i}^{\left(0\right)}\right)}^{2}$. In this section, the subscripts i , j and k are used as indices for the time variables $t\;,\;\;t_{1}$ and $t_{2}$, respectively.

For the first kernel $x_{}^{\left(1\right)}$ Equation (7), we get

(10) $$x_{i+1,j}^{\left(1\right)}=\left(1+a\Delta t\right)x_{i,j}^{\left(1\right)}-2\varepsilon \Delta tx_{i}^{\left(0\right)}x_{i,j}^{\left(1\right)}-4\varepsilon \Delta tI_{12}+\lambda \delta_{ij}x_{i}^{\left(0\right)},$$

where $I_{12}=\int_{R}x_{}^{\left(1\right)}\left(t_{1}\right)x_{}^{\left(2\right)}\left(t_{1},t_{2}\right)\;dt_{2}$. In the current work, the integrals $I_{1}\;,\;\;I_{2}$ and $I_{12}$ are computed using the midpoint integration rule.

For the second kernel $x_{}^{\left(2\right)}$ Equation (8), we get

(11) $$x_{i+1,j,k}^{\left(2\right)}=x_{i,j,k}^{\left(2\right)}+a\Delta tx_{i,j,k}^{\left(2\right)}-2\varepsilon \Delta tx_{i}^{\left(0\right)}x_{i,j,k}^{\left(2\right)}-\varepsilon \Delta tx_{i,j}^{\left(1\right)}x_{i,k}^{\left(1\right)}+\lambda \delta_{ik}x_{i,j}^{\left(1\right)}.$$

The time step Δt should be used to guarantee convergence of System (9)–(11). Applying the convergence criteria of the fixed-point iteration by differentiating right-hand side of Equation (9) with respect to $x_{}^{\left(0\right)}$ after neglecting *I***_1_** and *I*_2_ compared with ${\left(x_{i}^{\left(0\right)}\right)}^{2}$ to get

$$\begin{array}{l} 1+a\;\Delta t-2\varepsilon \;\Delta t\;x_{i}^{\left(0\right)}<0\;\;\;\;\;\to \;\;\;\;\;\left|a\;\Delta t-2\varepsilon \;\Delta t\;x_{i}^{\left(0\right)}\right|<1\\ ∴\;\;\Delta t<{\left|a\;-2\varepsilon \;x_{i}^{\left(0\right)}\right|}^{-1}\to \Delta t<\max{\left|a\;-2\varepsilon \;x_{i}^{\left(0\right)}\right|}^{-1}\to \Delta t<\varepsilon^{-1}{\left|\frac{a}{\varepsilon }\;-2\;x_{i}^{\left(0\right)}\right|}^{-1}\\ \to \Delta t<\varepsilon^{-1}{\left|L\;-2\;x_{i}^{\left(0\right)}\right|}^{-1}\to \Delta t<\varepsilon^{-1}L^{-1}\to \;\Delta t<1/a\\ \mathrm{Note}:\;\max\left[x_{i}^{\left(0\right)}\right]=a/\varepsilon =L:\mathrm{Carrying}\;\mathrm{Capacity} \end{array}$$

The condition Δt<1/a is a sufficient condition for the time step used in FDM for convergence. Due to similarity in the other two Equations (10) and (11), the same condition can be also used in the numerical FDM approximations for $x_{}^{\left(1\right)}$ and $x_{}^{\left(2\right)}$.

The mean and variance will be computed as

$$E\left[X\right]=x^{\left(0\right)}\;\mathrm{and}\;Var\left[X\right]=\sum_{k=1}^{M}\left(k!\right)\int_{R^{k}}{\left(x_{}^{\left(k\right)}\right)}^{2}d\tau_{k}=I_{1}+2I_{2},$$

where

$$I_{1}=\int_{R}{\left(x_{}^{\left(1\right)}\right)}^{2}dt_{1}\;\mathrm{and}\;\;I_{2}=2\int_{R^{2}}{\left(x_{}^{\left(2\right)}\right)}^{2}dt_{1}dt_{2}.$$

The integral $I_{1}$ represents the Gaussian part of the model variance and $I_{2}$ is the nonGaussian part of the total variance.

In the current work, the numerical simulations will consider the case of $a>\lambda^{2}/2$ which should converge, as descried above in Section 4, to the stationary gamma distribution. Other values of the parameter a are analyzed similarly as will be shown below.

**Figure 2:**
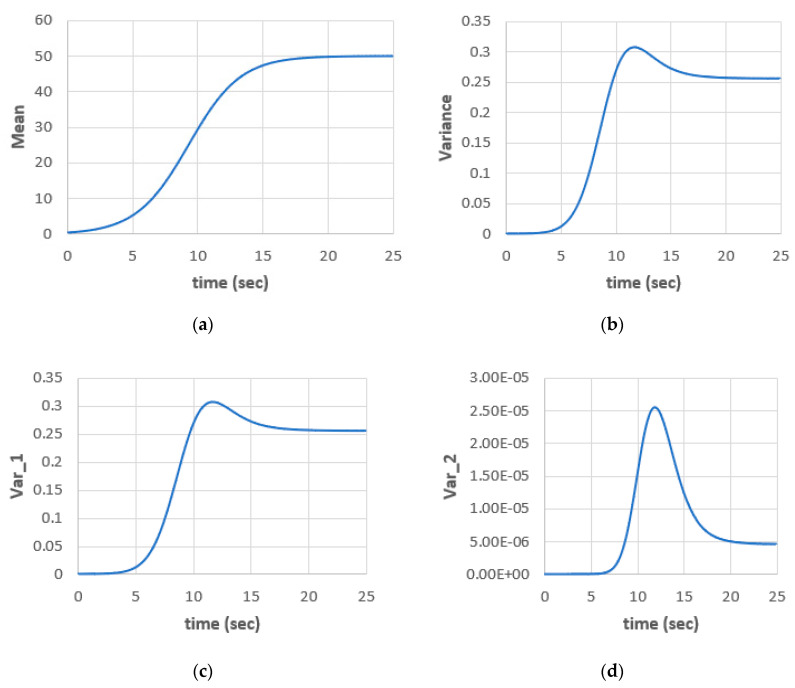
The stochastic population model; (**a**) mean; (**b**) total variance; (**c**) variance 1 (Gaussian only); (**d**) variance 2 (nonGaussian) for (Δt = 0.05, a = 0.5, ε = 0.01, λ = 0.01).

Figure 2 shows the time variation of the mean kernel $x_{}^{\left(0\right)}$ and the variance components: total variance, Var_1 = $I_{1}$ and Var_2 = $I_{2}$. The convergence of the kernels to steady-states is clear in the figure for the given parameters (Δt = 0.05, a = 0.5, ε = 0.01, λ = 0.01). The steady-state values for mean, total variance, Var_1 and Var_2 are 49.98, 0.2566, 0.2565 and 4.6 × 10^−6^, respectively. The nonGaussian part of the variance is small for the given parameters. The decay of the kernels is a known property and one of the known advantages of WHE.

**Figure 3:**
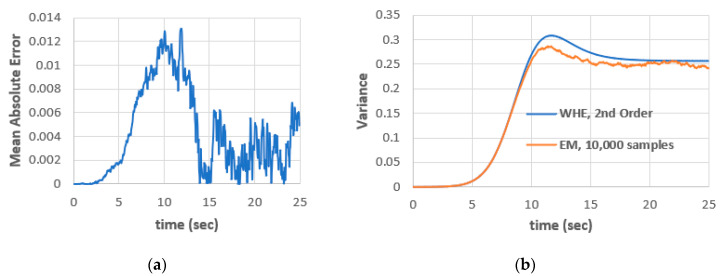
(**a**) Mean absolute error between EM (10,000 samples) and second-order WHE and (**b**) variance using EM (10,000 samples) and second-order WHE.

To validate the results, EM technique with 10,000 samples is considered. Figure 3 shows a comparison between EM and second-order WHE. The EM requires large number of samples to get a smooth solution and hence it will be of low efficiency. The convergence rate of EM is inversely proportional to the square root of number of samples. This issue makes EM insufficient when analyzing nonlinear and/or nonGaussian stochastic processes compared with WHE. The second-order WHE is used efficiently to simulate the dynamics in this case.

**Figure 4:**
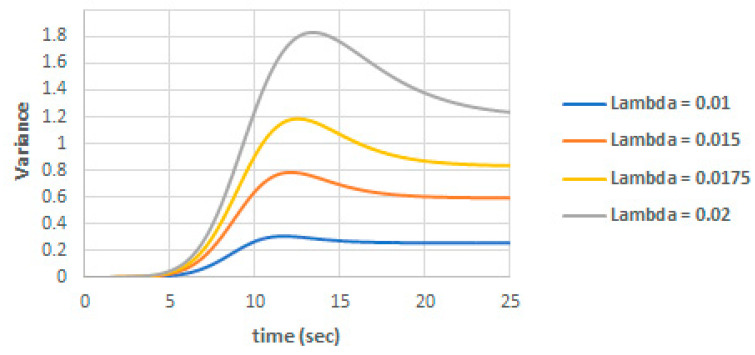
Variance for different values of λ.

The effect of λ on the total variance is shown in Figure 4. We can note the direct proportional relation between λ and the total variance.

**Figure 5:**
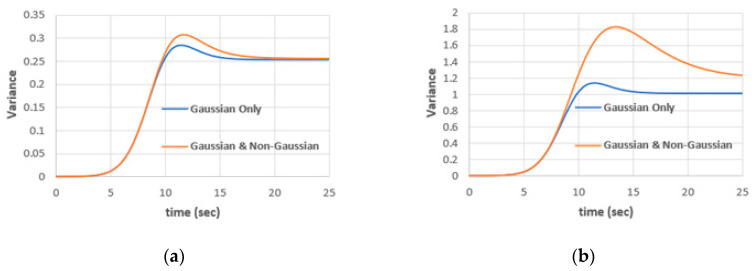
Gaussian variance and total variance (Gaussian with nonGaussian) (**a**) for λ = 0.01, (**b**) for λ = 0.02, (Δt = 0.05, a = 0.5, ε = 0.01).

To quantify the effect of the parameter λ, Figure 5 shows the variance, Gaussian only and Gaussian with nonGaussian, for λ = 0.01 and λ = 0.02. We can note that as λ increases, the nonGaussian variance and hence the total variance increases as well. This reflects the nonlinearity, and hence the nonGaussian nature of the population model. The model is sensitive to the value of λ. From Figure 5 we can estimate the nonGaussian effect compared with the Gaussian response of the model. For λ = 0.01, the nonGaussian variance contribution is only 1.4% of the total variance, while for λ = 0.02, the nonGaussian variance contribution is 20.4% of the total variance. Estimating the nonGaussian variance contribution is an advantage in WHE over the EM and sampling techniques.

**Figure 6:**
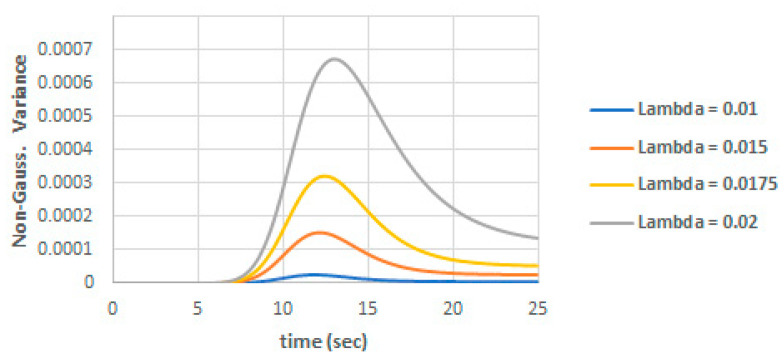
NonGaussian variance component for different values of λ.

The nonGaussian part of the variance is shown in Figure 6 against λ. We can note that the nonGaussian part of the variance increases to a peak value near the inflection of the mean population and then start to decay to a uniform value. This can be explained as the system dynamics is nonlinear and severe no-Gaussian around the curve inflection.

**Figure 7:**
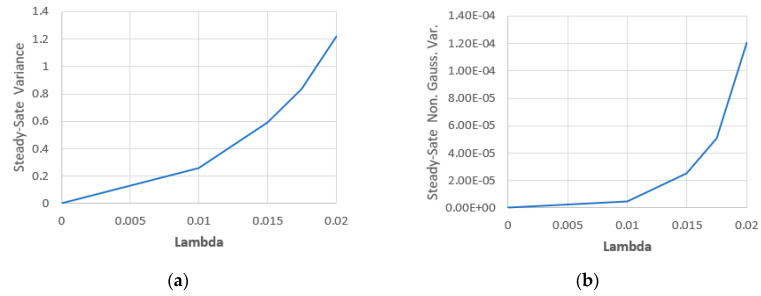
(**a**) Steady-state total variance with λ and (**b**) the steady-state nonGaussian part of variance with λ.

The steady-state variance components with different values of λ are shown in Figure 7 and Table 1. We can note, by interpolation, that the steady-state total variance is proportional to $\lambda^{2}$ while the steady-state nonGaussian variance component is proportional to $\lambda^{4}$.

## 6. Stochastic Model with Random Parameters

In the case of random variation of one or more of the model parameters, the variance will increase when compared with the case of only noise as the source of randomness. The kernels $x^{\left(j\right)};j\leq M$ can be further expanded/decomposed using gPC. For example, we assume the parameter ***a*** is a random parameter that depends on a set of standard random variables with known distribution. This means we can write

$$a\left(\omega \right)=\sum_{k=0}^{P}a_{k}\;\psi_{k},$$

where the subscript *k* is the index of gPC term. Then, the kernels $x^{\left(j\right)};j\leq M$ are decomposed as

$$x^{\left(j\right)}=\sum_{k=0}^{P}x_{k}^{\left(j\right)}\;\psi_{k}\;\;;\;\;\;j\leq M$$

with $x_{0}^{\left(j\right)}$ as the mean and $\sum_{k=1}^{P}{\left[x_{k}^{\left(j\right)}\right]}^{2}\;$ as the variance of $x^{\left(j\right)};j\leq M$.

Substitute in $x_{}^{\left(0\right)}$ Equation (6) to get

(12) $$\begin{array}{ll} \frac{d}{dt}\sum_{k=0}^{P}x_{k}^{\left(0\right)}\psi_{k}=a\;\sum_{k=0}^{P}x_{k}^{\left(0\right)}\psi_{k}\;-\;\varepsilon \;{\left(\sum_{k=0}^{P}x_{k}^{\left(0\right)}\psi_{k}\right)}^{2} & \;-\;\varepsilon \int_{R}{\left(\sum_{k=0}^{P}x_{k}^{\left(1\right)}\psi_{k}\right)}^{2}dt_{1}\\ & -\;2\varepsilon \;\int_{R^{2}}{\left(\sum_{k=0}^{P}x_{k}^{\left(2\right)}\psi_{k}\right)}^{2}dt_{1}dt_{2} \end{array}$$

Multiply Equation (12) by $\psi_{k}\;;\;\;k\geq 0$ and take the average with respect to gPC basis to get

$$\begin{array}{ll} \frac{d}{dt}\sum_{k=0}^{P}x_{k}^{\left(0\right)} & \langle \psi_{k}\psi_{j}\rangle =a\;\sum_{k=0}^{P}x_{k}^{\left(0\right)}\langle \psi_{k}\psi_{j}\rangle \;-\;\varepsilon \sum_{k=0}^{P}\sum_{i=0}^{P}x_{i}^{\left(0\right)}x_{k}^{\left(0\right)}\langle \psi_{i}\psi_{k}\psi_{j}\rangle \;\\ & -\;\varepsilon \int_{R}\sum_{k=0}^{P}\sum_{i=0}^{P}x_{i}^{\left(1\right)}x_{k}^{\left(1\right)}\langle \psi_{i}\psi_{k}\psi_{j}\rangle \;dt_{1}-\;2\varepsilon \;\int_{R^{2}}\sum_{k=0}^{P}\sum_{i=0}^{P}x_{i}^{\left(2\right)}x_{k}^{\left(2\right)}\langle \psi_{i}\psi_{k}\psi_{j}\rangle \;dt_{1}dt_{2} \end{array}$$

To derive the equivalent deterministic system, we need the expected values for the products $\langle \psi_{k}\psi_{j}\rangle$ and $c_{ijk}=\langle \psi_{i}\;\psi_{j}\;\psi_{k}\rangle$. From the orthogonality property of functionals $\psi_{k}\;;\;\;k\geq 0$, we use $\langle \psi_{k}\psi_{j}\rangle =\delta_{ij}$, where δ is the Kronecker delta function. Details about computing expectations $c_{ijk}$ can be found in [20]. Similar expressions for $x_{}^{\left(1\right)}$ and $x_{}^{\left(2\right)}$ are straight-forward.

In the case where the parameter a depends only one random variable $\psi_{1}$, we can write $a\left(\omega \right)=a_{0}+a_{1}\psi_{1}$. This yields the following for $x^{\left(0\right)}$:

$$\frac{dx_{0}^{\left(0\right)}}{dt}=\left(a_{0}x_{0}^{\left(0\right)}+a_{1}x_{1}^{\left(0\right)}\;-\;\varepsilon \;\left(\begin{array}{l} \;{\left(x_{0}^{\left(0\right)}\right)}^{2}\;+\int_{R}{\left(x_{0}^{\left(1\right)}\right)}^{2}dt_{1}+2\;\int_{R^{2}}{\left(x_{0}^{\left(2\right)}\right)}^{2}dt_{1}dt_{2}\\ +{\left(x_{1}^{\left(0\right)}\right)}^{2}\;+\int_{R}{\left(x_{1}^{\left(1\right)}\right)}^{2}dt_{1}+2\;\int_{R^{2}}{\left(x_{1}^{\left(2\right)}\right)}^{2}dt_{1}dt_{2} \end{array}\right)\right),\;\frac{dx_{1}^{\left(0\right)}}{dt}=\left(a_{0}x_{1}^{\left(0\right)}+a_{1}x_{0}^{\left(0\right)}\;-\;2\varepsilon \;\left(\;x_{0}^{\left(0\right)}x_{1}^{\left(0\right)}\;+\int_{R}x_{0}^{\left(1\right)}x_{1}^{\left(1\right)}dt_{1}+2\;\int_{R^{2}}x_{0}^{\left(2\right)}x_{1}^{\left(2\right)}dt_{1}dt_{2}\right)\right).$$

Similarly, for $x^{\left(1\right)}$:

$$\frac{dx_{0}^{\left(1\right)}}{dt}=\left(a_{0}x_{0}^{\left(1\right)}+a_{1}x_{1}^{\left(1\right)}\;-\;\varepsilon \;\left(\begin{array}{l} 2\;x_{0}^{\left(0\right)}x_{0}^{\left(1\right)}+\;4\int_{R}x_{0}^{\left(1\right)}\left(t_{1}\right)x_{0}^{\left(2\right)}\left(t_{1},t_{2}\right)\;dt_{2}\\ +2\;x_{1}^{\left(0\right)}x_{0}^{\left(1\right)}+\;4\int_{R}x_{1}^{\left(1\right)}\left(t_{1}\right)x_{1}^{\left(2\right)}\left(t_{1},t_{2}\right)\;dt_{2} \end{array}\right)\right)+\lambda \;\delta (t-t_{1})x_{0}^{\left(0\right)}\;\frac{dx_{1}^{\left(1\right)}}{dt}=\left(a_{0}x_{1}^{\left(1\right)}+a_{1}x_{0}^{\left(1\right)}\;-\;2\varepsilon \;\left(\begin{array}{l} \;\;\;\;x_{0}^{\left(0\right)}x_{1}^{\left(1\right)}+\;2\int_{R}x_{0}^{\left(1\right)}\left(t_{1}\right)x_{1}^{\left(2\right)}\left(t_{1},t_{2}\right)\;dt_{2}\\ +\;x_{1}^{\left(0\right)}x_{0}^{\left(1\right)}+\;2\int_{R}x_{1}^{\left(1\right)}\left(t_{1}\right)x_{0}^{\left(2\right)}\left(t_{1},t_{2}\right)\;dt_{2} \end{array}\right)\right)+\lambda \;\delta (t-t_{1})x_{1}^{\left(0\right)}$$

and for $x^{\left(2\right)}$:

$$\frac{dx_{0}^{\left(2\right)}}{dt}=\left(a_{0}x_{0}^{\left(2\right)}+a_{1}x_{1}^{\left(2\right)}\;-\;\varepsilon \;\left(\begin{array}{l} \;2x_{0}^{\left(0\right)}x_{0}^{\left(2\right)}+x_{0}{}^{\left(1\right)}\left(t_{1}\right)x_{0}{}^{\left(1\right)}\left(t_{2}\right)\\ +2x_{1}^{\left(0\right)}x_{1}^{\left(2\right)}+x_{1}{}^{\left(1\right)}\left(t_{1}\right)x_{1}{}^{\left(1\right)}\left(t_{2}\right) \end{array}\right)\right)+\lambda \;\delta (t-t_{2})x_{0}^{\left(1\right)}\;\frac{dx_{1}^{\left(2\right)}}{dt}=\left(a_{0}\;x_{1}^{\left(2\right)}+a_{1}\;x_{0}^{\left(2\right)}\;-\;2\varepsilon \left(x_{0}^{\left(0\right)}x_{1}^{\left(2\right)}+x_{1}^{\left(0\right)}x_{0}^{\left(2\right)}+x_{0}{}^{\left(1\right)}\left(t_{1}\right)x_{1}{}^{\left(1\right)}\left(t_{2}\right)\right)\right)+\lambda \;\delta (t-t_{2})x_{1}^{\left(1\right)}$$

When considering only the Gaussian part of the solution with only one random parameter, the system will be reduced to the following:

$$\frac{dx_{0}^{\left(0\right)}}{dt}=\left(a_{0}x_{0}^{\left(0\right)}+a_{1}x_{1}^{\left(0\right)}\;-\;\varepsilon \;\left(\;{\left(x_{0}^{\left(0\right)}\right)}^{2}\;+{\left(x_{1}^{\left(0\right)}\right)}^{2}+\int_{R}\left[{\left(x_{0}^{\left(1\right)}\right)}^{2}+{\left(x_{1}^{\left(1\right)}\right)}^{2}\right]dt_{1}\right)\right),\;\frac{dx_{0}^{\left(1\right)}}{dt}=\left(a_{0}x_{0}^{\left(1\right)}+a_{1}x_{1}^{\left(1\right)}\;-\;2\varepsilon \;\left(x_{0}^{\left(0\right)}x_{0}^{\left(1\right)}+\;x_{1}^{\left(0\right)}x_{0}^{\left(1\right)}\right)\right)+\lambda \;\delta (t-t_{1})x_{0}^{\left(0\right)},\;\frac{dx_{1}^{\left(0\right)}}{dt}=\left(a_{0}x_{1}^{\left(0\right)}+a_{1}x_{0}^{\left(0\right)}\;-\;2\varepsilon \;\left(\;x_{0}^{\left(0\right)}x_{1}^{\left(0\right)}\;+\int_{R}x_{0}^{\left(1\right)}x_{1}^{\left(1\right)}dt_{1}\right)\right),\;\frac{dx_{1}^{\left(1\right)}}{dt}=\left(a_{0}x_{1}^{\left(1\right)}+a_{1}x_{0}^{\left(1\right)}\;-\;2\varepsilon \;\left(x_{0}^{\left(0\right)}x_{1}^{\left(1\right)}+\;x_{1}^{\left(0\right)}x_{0}^{\left(1\right)}\right)\right)+\lambda \;\delta (t-t_{1})x_{1}^{\left(0\right)}.$$

Similar expressions can be obtained for the random variation in the parameter ε.

We can summarize the above decomposition in the case of combined noise and random parameters with the expression

$$X(t;\omega )=\sum_{j=0}^{P}\sum_{k=0}^{M}\int_{Rk}x_{j}^{\left(k\right)}(t)\;\psi_{j}H^{\left(k\right)}d\tau_{k}.$$

Which can be applied in any order either WHE-gPC or gPC-WHE. Using WHE-gPC is more efficient and results in simpler deterministic system.

In the above derivation, we assumed independency between noise and the random parameters. This happens usually in cases of uncertainties occurring from different uncorrelated sources. In this case, the statistical properties, mean and total variance, are computed as follows:

$$E[X]=x_{0}^{\left(0\right)}\;\mathrm{and}\;Var\left[X\right]=\sum_{j=0}^{P}\sum_{\begin{array}{l} \;\;\;\;\;\;\;\;k=0\\ \;\left(j,k\right)\neq \left(0,0\right) \end{array}}^{M}k!\int_{R^{j}}{\left(x_{j}^{\left(k\right)}\right)}^{2}d\tau_{k}.$$

The total variance Var[X] can be analyzed/decomposed into three components: variance $Var_{par}$ due to random parameters, variance $Var_{noise}$ due to noise and variance $Var_{mix}$ due to the mix between noise and the random parameters, i.e.,

$$Var\left[X\right]=Var_{par}+Var_{noise}+Var_{mix},$$

where

$$Var_{par}=\sum_{k=1}^{P}{\left[x_{k}^{\left(0\right)}\right]}^{2},\;Var_{noise}=\sum_{k=1}^{M}k!\int_{R^{k}}{\left[x_{0}^{\left(k\right)}\right]}^{2}d\tau_{k},\;Var_{mix}=\sum_{j=1}^{P}\sum_{\;k=1}^{M}k!\int_{R^{k}}{\left[x_{j}^{\left(k\right)}\right]}^{2}d\tau_{k}.$$

In the case where only one random parameter in the model SDE and only the Gaussian solution is to be considered, the variance components will be as follows:

$$Var_{par}={\left[x_{1}^{\left(0\right)}\right]}^{2},\;Var_{noise}=\int_{R^{}}{\left[x_{0}^{\left(1\right)}\right]}^{2}dt_{1},\;Var_{mix}=\int_{R^{}}{\left[x_{1}^{\left(1\right)}\right]}^{2}dt_{1}.$$

In the case of dependency between the random parameters and/or the noise, the same above derivation can be extended after considering the covariance between the dependent sources. Alternatively, the WCE technique can be used at which the noise is approximated by a set of random variables. In this case, the system will be affected only by uncertainties due to a set of random parameters [21]. The drawback of using WCE is the low convergence due to the noise approximation by a few number of random variables. Using WHE is efficient compared with many other techniques [22].

## 7. Numerical Example with Combined Randomness

**Figure 8:**
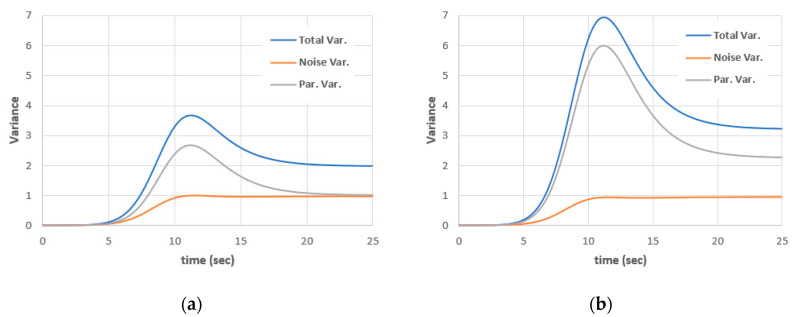
Variance components for λ = 0.02, ε = 0.01, (**a**) in case of $a\left(\omega \right)=0.5+0.01\;\;\psi_{1}$ and (**b**) in case of $a\left(\omega \right)=0.5+0.015\;\;\psi_{1}$.

Assume that the parameter ***a*** fluctuates uniformly around the mean with deviation 2% of the mean value $a_{0}$, i.e., $a=0.5+0.01\;\;\psi_{1}$ with $\psi_{1}∼U[-1,\;1]$. This is equivalent to a uniform distribution a∼U[0.49,  0.51]. Using λ = 0.02, ε = 0.01 and zero initial conditions for all kernels except for $x_{0}^{\left(0\right)}(t=0)$ = 0.5. The total variance and its components, due to noise and due to random parameters, are shown Figure 8. The variance due to mixed contributions are very small compared with other variance components. It is four orders of magnitude smaller than other variance components and hence will be neglected in the analysis.

For 2% deviations, the steady-state total variance is 1.982, while it is 1.013 in the case of the deterministic parameter *a*,. This means that 2% deviations in *a* result in 95% deviation in the steady-state total variance. For 3% deviations, the steady-state variance is 3.22, while it is 1.013 in the case of the deterministic parameter *a*. This means that 3% deviations in *a* result in 218% deviation in the steady-state total variance. The model is very sensitive to the deviations in parameter *a*.

The system sensitivity indices [15], due to different sources of randomness, can be estimated from the variance components. The sensitivity index is the ratio of the variance component to the total variance. For example, the sensitivity index $S_{noise}$ due to noise is calculated as

$$S_{noise}=Var_{noise}/Var[X].$$

**Figure 9:**
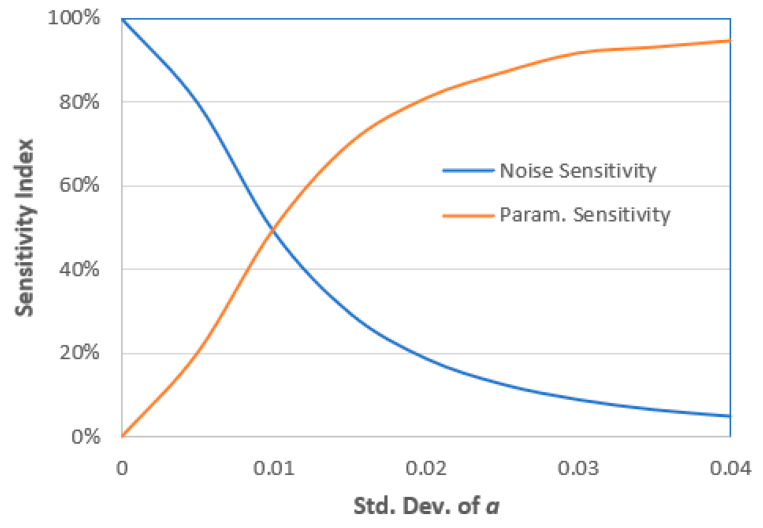
Sensitivity indices due to noise and random parameter *a*.

The sensitivity indices due to random parameter *a* and due to noise are shown in Table 2. The model is 100% sensitive to noise in the case of the parameter *a*, which is deterministic. Any deviations in the parameter ***a*** will affect the system sensitivity indices. As the deviations in ***a*** increase, the system response deviates further (i.e., variance increases) and becomes more sensitive toward the deviations in ***a***. This result is also shown in Figure 9, which compares the sensitivity indices for a wide range of a deviations.

**Figure 10:**
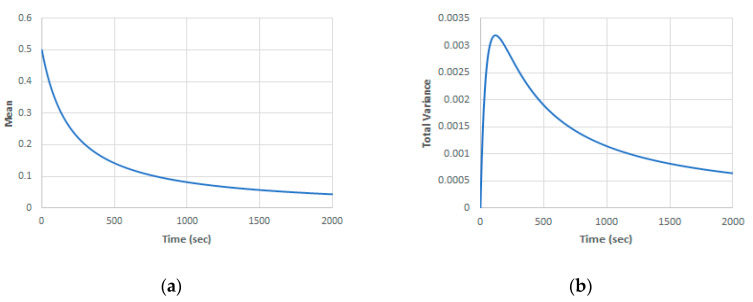
Case of $a<\lambda^{2}/2$ ($a\left(\omega \right)=0.0001+0.00004\;\;\psi_{1}$, λ = 0.02, ε = 0.01, (**a**) mean solution; (**b**) total variance.

To test different scenarios, the developed system with mixed uncertainties is simulated in the case of $a<\lambda^{2}/2$ where the population extinction is expected. Figure 10 shows the mean and total variance in the case of $a\left(\omega \right)=0.0001+0.00004\;\;\psi_{1}$, λ = 0.02, ε = 0.01 and zero initial conditions for all kernels except for $x_{0}^{\left(0\right)}(t=0)$ = 0.5. In this case, the main population decays slowly to zero and the variance will be mainly due to noise (more than 99.9% of the total variance). This means the model is not sensitive to the random parameter variations in this case.

The above analysis can be extended to many random parameters in addition to the noise imposed to the model. In all cases, we shall need only to solve a system of few number of deterministic equations that can be analyzed with the available well-known analytical and/or numerical techniques. This gives a great advantage over the sampling techniques that require a huge number of runs to estimate reliable statistics of the model under consideration.

## 8. Conclusions

In this paper, a decomposition technique based on spectral random and stochastic-combined expansion is considered. The combined expansion is a tool that can be applied to models under noise and random parameter variations such as the population growth model. The introduced technique can be applied sequentially in any order to derive an equivalent system that is deterministic and can be analyzed using the well-known analytical and/or numerical techniques.

The stochastic population model is analyzed using WHE, and the model statistics are obtained using the statistical properties of WHE basis functions. The gPC decomposition technique is then applied to analyze the effect of the random parameters such as the growth rate. The proposed technique allows us to decompose the total variance into components due to noise and due to random parameters. This allows for estimating the model sensitivity due to different sources of randomness. The proposed technique can be extended to allow for analyzing models with many sources of randomness such as the population models.

## Figures and Tables

**Table 1:** Steady-state variance components with λ.

λ	Total Var.	NonGaussian Var.
0.0000	0.000	0
0.0100	0.257	4.59 × 10^−6^
0.0150	0.593	2.50 × 10^−5^
0.0175	0.835	5.10 × 10^−5^
0.0200	1.220	1.21 × 10^−4^

**Table 2:** Sensitivity indices due to noise and the random parameter ***a***.

Parameter a	Noise Sensitivity Index	Parameter a Sensitivity Index
a(ω)=0.5 (deterministic)	100%	0.0%
$a\left(\omega \right)=0.5+0.005\;\;\psi_{1}$	79.8%	20.2%
$a\left(\omega \right)=0.5+0.010\;\;\psi_{1}$	49.1%	49.9%
$a\left(\omega \right)=0.5+0.015\;\;\psi_{1}$	29.7%	70.3%
$a\left(\omega \right)=0.5+0.020\;\;\psi_{1}$	18.9%	81.1%
$a\left(\omega \right)=0.5+0.025\;\;\psi_{1}$	12.8%	87.2%
$a\left(\omega \right)=0.5+0.030\;\;\psi_{1}$	9.1%	91.9%
$a\left(\omega \right)=0.5+0.035\;\;\psi_{1}$	6.7%	93.3%
